# Four-year effects of exercise on fatigue and physical activity in patients with cancer

**DOI:** 10.1186/s12916-018-1075-x

**Published:** 2018-06-08

**Authors:** Lenja Witlox, Anouk E. Hiensch, Miranda J. Velthuis, Charlotte N. Steins Bisschop, Maartje Los, Frans L. G. Erdkamp, Haiko J. Bloemendal, Marlies Verhaar, Daan ten Bokkel Huinink, Elsken van der Wall, Petra H. M. Peeters, Anne M. May

**Affiliations:** 1 Department of Clinical Epidemiology, Julius Center for Health Sciences and Primary Care, University Medical Center Utrecht, Utrecht University, PO Box 85500, STR 6.131, 3508 GA Utrecht, The Netherlands; 2 Netherlands Comprehensive Cancer Organisation (IKNL), PO Box 19079, 3501 DB Utrecht, The Netherlands; 3 Department of Medical Oncology, St. Antonius Ziekenhuis, Koekoekslaan 1, 3435 CM Nieuwegein, The Netherlands; 4 Department of Internal Medicine - Medical Oncology, Obis Medisch Centrum, Dr van der Hoffplein 1, 6166 BG Sittard-Geleen, The Netherlands; 5 Department of Internal Medicine, Meander Medical Center, Maatweg 3, 3818 TZ Amersfoort, The Netherlands; 6 Department of Internal Medicine, Hofpoort Ziekenhuis, Polanerbaan 2, 3447 GN Woerden, The Netherlands; 7 Department of Internal Medicine, Diakonessenhuis, Bosboomstraat 1, 3582 KE Utrecht, The Netherlands; 8 Department of Medical Oncology, University Medical Center Utrecht, Utrecht University, Heidelberglaan 100, 3584 CX Utrecht, The Netherlands

**Keywords:** Cancer, Exercise intervention, Chemotherapy, Fatigue, Physical activity, Long-term effects

## Abstract

**Background:**

In the earlier randomized controlled Physical Activity during Cancer Treatment (PACT) study, we found beneficial effects of an 18-week supervised exercise program on fatigue in patients with newly diagnosed breast or colon cancer undergoing adjuvant treatment. The present study assessed long-term effects of the exercise program on levels of fatigue and physical activity 4 years after participation in the PACT study.

**Methods:**

The original study was a two-armed, multicenter randomized controlled trial comparing an 18-week supervised exercise program to usual care among 204 breast cancer patients and 33 colon cancer patients undergoing adjuvant treatment. Of the 237 PACT participants, 197 participants were eligible and approached to participate in the 4-year post-baseline measurements, and 128 patients responded. We assessed fatigue and physical activity levels at 4 years post-baseline and compared this to levels at baseline, post-intervention (18 weeks post-baseline), and at 36 weeks post-baseline.

**Results:**

Intention-to-treat mixed linear effects model analyses showed that cancer patients in the intervention group reported significantly higher moderate-to-vigorous total physical activity levels (141.46 min/week (95% confidence interval (CI) 1.31, 281.61, effect size (ES) = 0.22) after 4 years compared to the usual care group. Furthermore, cancer patients in the intervention group tended to experience less physical fatigue at 4 years post-baseline compared to the usual care group (− 1.13, 95% CI –2.45, 0.20, ES = 0.22), although the result was not statistically significant.

**Conclusion:**

Patients with breast or colon cancer who participated in the 18-week exercise intervention showed significant higher levels of moderate-to-vigorous total physical activity levels and a tendency towards lower physical fatigue levels 4 years post-baseline. Our result indicate that exercising during chemotherapy is a promising strategy for minimizing treatment-related side effects, both short and long term.

**Trial registration:**

Current Controlled Trials ISRCTN43801571, Dutch Trial Register NTR2138. Trial registered on 9 December 2009.

## Background

Cancer-related fatigue is the most common and distressing treatment-related side effect. It may persist for many years and result in impairment of quality of life and physical functioning [1–3]. Increasing evidence suggests that exercise interventions delivered during adjuvant cancer treatment have beneficial short-term effects on fatigue in cancer patients [4, 5].

Between 2010 and 2013, we performed the randomized controlled Physical Activity during Cancer Treatment (PACT) study and found lower levels of physical fatigue after an 18-week supervised exercise intervention delivered during adjuvant treatment in patients with breast or colon cancer [6, 7]. The PACT intervention included both aerobic and resistance training at a moderate-to-high intensity and also incorporated principles of Bandura’s social cognitive theory to help participants maintain a physically active lifestyle after completion of the exercise program [8]. Maintaining a physically active lifestyle into survivorship might positively influence fatigue levels in the long term.

Since there have been notable improvements in cancer survival rates, and cancer treatment is known to have long-lasting side effects including fatigue, it is important to gain more insight into the potential effects of exercising during treatment on fatigue years after completion of adjuvant cancer treatment [4, 9, 10]. Most randomized controlled trials (RCTs) followed participants up to 6 months post-intervention and showed trends of decreased fatigue in favor of the exercise intervention group [11–13]. Only one intervention study followed previous breast cancer study participants for 5 years. The researchers found that women in the intervention group reported more time spent in leisure-time activities, more periods with positive mood, and more favorable motivational outcomes compared to the control group [14, 15]. Long-term effects on fatigue were not assessed.

Therefore, the aim of the present study is to assess long-term effects of the PACT exercise intervention on fatigue (the primary outcome of the original PACT study) and physical activity levels. These measurements were taken on average 4 years after enrollment in the PACT study.

## Methods

### Settings and participants

A detailed description of the PACT study design has been published previously [16]. The original study was conducted in seven hospitals in the Netherlands between 2010 and 2013. In short, this multicenter controlled trial randomly assigned 204 breast and 33 colon cancer patients to either usual care (*n* = 118) or to supervised aerobic and muscle strength training in addition to usual care (*n* = 119). Inclusion criteria were as follows: a histological diagnosis of cancer less than 6 weeks (breast cancer) or 10 weeks (colon cancer) before study recruitment; stage M0; scheduled for chemotherapy; age 25–75 years; not treated for any cancer in the preceding 5 years (except basal skin cancer); able to read and understand the Dutch language; Karnovsky Performance Status of ≥60; able to walk 100 m or more; and no contraindications for physical activity (as assessed through the Revised Physical Activity Readiness Questionnaire). After written informed consent was obtained, a concealed computer-generated randomization, following a 1:1 ratio and stratified for age (25–40, 40–65, and 65–75 years), adjuvant treatment (radiotherapy yes/no before chemotherapy), use of tissue expander (for breast cancer patients yes/no), tumor type, and hospital, was used to allocate patients to the two groups. The study was approved by the Medical Ethics Committee of the University Medical Center Utrecht and the local ethical boards of the participating hospitals.

**Fig. 1:**
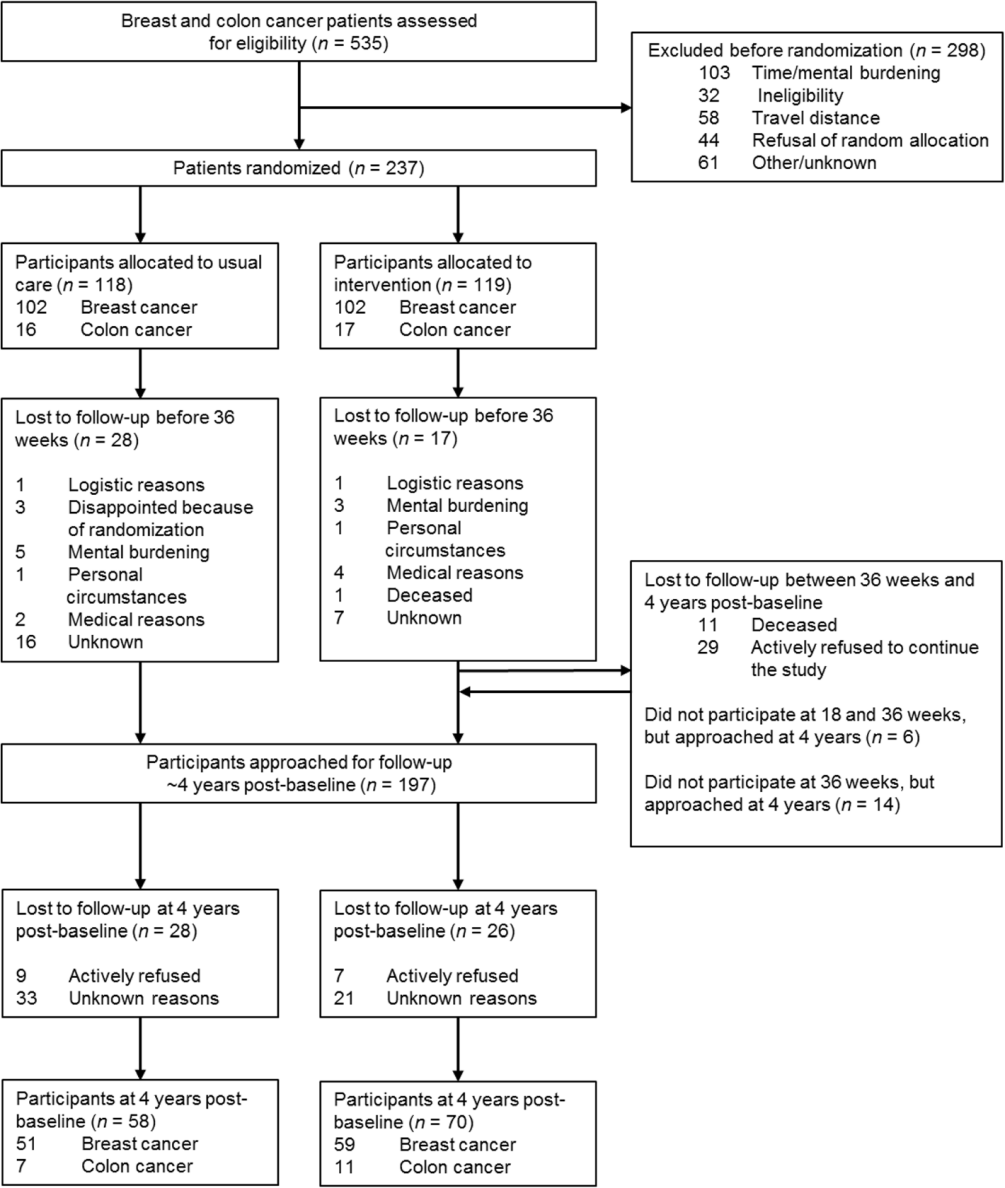
Participant flow through the study

Three to four years after inclusion in the original PACT study, the treating physicians approached 197 PACT participants again for information on their current health status. The present study was originally not planned when participants were recruited for the PACT study, and consent was asked again. Eleven participants were deceased or otherwise considered not healthy enough to participate by the treating oncologist (Fig. 1). We did not invite participants who dropped out of the intervention and/or did not perform the evaluation measurements during the original PACT study and also indicated they were not willing to complete questionnaires at subsequent time points. We also excluded participants who were not willing to be invited to future research (*n* = 28). Participants who dropped out during the original PACT study but indicated they were willing to complete questionnaires at all subsequent time points were invited. Patients who signed written informed consent were asked to complete questionnaires at home.

### Intervention

The exercise intervention consisted of an 18-week supervised aerobic and muscle strength exercise program in addition to the usual care. Twice a week participants attended a 1-h session supervised by a physiotherapist. The aerobic and muscle strength exercises were individualized to the participants’ preferences and fitness levels as assessed by a cardiopulmonary exercise test and a one-repetition maximum muscle strength test. In addition to the intervention, participants were asked to be physically active for at least 30 min a day on 3 other days of the week according to the Dutch guideline for physical activity [17]. Principles of Bandura’s social cognitive theory were incorporated to promote maintenance of a physically active lifestyle [8]. This theory emphasizes the role of cognitive processes in determining behavior such as exercise. The most important construct in this theory is self-efficacy, which can be altered by mastery experience, vicarious or observational learning, and verbal persuasion. First, mastery experience was used by asking participants to report training results in graph form. Second, physiotherapists checked the graphs weekly, gave positive feedback about the obtained results, and stimulated the participant to make action plans to further increase their physical activity level*.*

Participants in the control group received the usual care and were asked to maintain their habitual physical activity pattern up to week 18. Thereafter they were allowed, for ethical reasons, to participate in exercise programs offered in the Netherlands to patients with cancer after completion of primary treatment.

## Outcome measures

The outcomes were assessed at baseline, post-intervention (18 weeks post-baseline), and at 36 weeks (post-baseline) in the original PACT study. In the present study, outcomes were measured at a median of 4 years post-baseline.

### Fatigue

Fatigue was assessed using the validated Dutch version of the Multidimensional Fatigue Inventory (MFI) [18]. The MFI is a 20-item questionnaire and consists of five dimensions: general fatigue, physical fatigue, mental fatigue, reduced activity, and reduced motivation. Scores of the subscales range from 4 to 20, and a higher score indicates higher levels of fatigue.

### Physical activity

Physical activity levels were assessed using the Short Questionnaire to Assess Health-enhancing physical activity (SQUASH) [19]. The validated four-item questionnaire contains questions about commuting, leisure time and sports, household activities, and activities at work and school. For each activity, duration, frequency, and intensity are assessed. At baseline, we asked participants to fill in their physical activity level for a usual week in the months preceding study entry. Minutes per week of moderate-to-high intensity total physical activity and leisure-time and sport activity were calculated. Moderate-to-high intensity physical activity was defined as ≥4 metabolic equivalent (METs).

### Statistical analysis

Sample size calculations were performed for the original PACT study with fatigue at 18 weeks (post-intervention) as the primary outcome. In order to detect a between-group change in fatigue of 2 units (±4 standard deviation (SD)), corresponding to a medium effect size (ES), and anticipating a drop-out of 10%, 75 participants in the intervention group and 75 participants in the usual care group were needed (α = 0.05, power = 0.80). Taking the correlation (ρ) between baseline and follow-up measurements into account by multiplying the previously calculated number of subjects by (1 – ρ^2^), plus one extra subject per group [20], yielded a sample size of 64 participants per group, which enables us to detect similar ESs. Note that here we analyzed data for breast and colon cancer patients together, in contrast to the original PACT study.

Baseline demographics were summarized for all breast and colon cancer patients together and compared for those who had and had not dropped out of the study 4 years post-baseline. Intention-to-treat mixed linear regression models were used to model fatigue and physical activity at 18 weeks, 36 weeks, and 4 years for all breast and colon cancer patients combined and for breast cancer patients only. These models were adjusted for the baseline value of the outcome and tumor receptor status (triple negative; Her2Neu+, estrogen receptor (ER)+ or progesterone receptor (PR)+; Her2Neu+, ER– and PR–; Her2Neu–, ER+ or PR+ (for breast cancer patients)) as well as for the stratification factors: age (25–40, 40–65, and 65–75), adjuvant treatment (radiotherapy yes/no before chemotherapy), hospital, tumor site, and use of tissue expander (for breast cancer patients yes vs. no). Between-group differences were modeled using outcome measurements obtained at 18 weeks (post-intervention), and 36 weeks and 4 years post-baseline. Within-group differences were modeled using outcome measurements obtained at all four time points (i.e., at baseline, 18 weeks, 36 weeks, and 4 years). Analyses were performed on an intention-to-treat basis for all 237 breast and colon cancer patients in the original PACT study with at least one measurement, except when this was the baseline value. The model accounts for missing data by taking the individual time trends and the observed group means at each time point into consideration to provide a more accurate estimate of the population mean at each time point.

Mean differences and 95% confidence intervals (CIs) were accompanied by standardized ESs. The standardized ESs were calculated by dividing the adjusted between-group difference of the 4-year post-baseline mean by the pooled baseline standard deviation. According to Cohen, ESs < 0.2 indicate “no difference,” whereas ESs between 0.2 and 0.5 indicate “small differences,” ESs between 0.5 and 0.8 indicate “medium and clinically relevant differences,” and ESs ≥0.8 indicate “large differences” [21]. Statistical significance was set at a probability of *p* < 0.05 for all analyses. Statistical analyses were performed using SPSS statistics version 21.0.

## Results

### Participants

Between 2010 and 2013, a total of 237 patients with breast or colon cancer were included in the original PACT study (Fig. 1). In total, 119 patients were randomized to the exercise intervention and 118 patients to the control group. Patients randomized to the intervention group attended 83% (interquartile range 69–91%) of the supervised sessions.

Four years post-baseline, 197 PACT participants were eligible and approached to participate in the 4 year post-baseline measurements, and finally 128 (65.0%) participants signed informed consent. Sixteen PACT participants refused to participate in the 4-year post-baseline measurements, and 54 PACT participants did not respond for unknown reasons, also after one reminder had been sent. Patient characteristics were not significantly different between participating patients and non-participating eligible patients (*p* > 0.05).

At baseline, characteristics of all original PACT participants in the intervention and the usual care group were comparable (Table 1), except that participants in the intervention group showed higher education (46.2% vs. 38.1%, respectively), had more frequent triple negative breast cancer (20.2% vs. 10.2%), and were more often post-menopausal (40.3% vs. 30.5%).

**Table 1:** Baseline characteristics of participants of the original PACT study (*n* = 237) and responders post-baseline (*n* = 128)

	All participants		Responders ~ 4 years post-baseline	
	Intervention *n* = 119	Usual care *n* = 118	Intervention *n* = 70	Usual care *n* = 58
Socio-demographical factors				
Age (years)	50.9 ± 9.0	50.6 ± 8.6	51.1 ± 8.3	51.6 ± 7.9
Sex, female	109 (91.6)	107 (90.7)	64 (91.4)	53 (91.4)
Marital status				
Couple	91 (76.5)	87 (73.7)	59 (84.3)	48 (82.8)
Single	24 (20.2)	25 (21.2)	11 (15.7)	10 (17.2)
Education				
Low	5 (4.2)	21 (17.8)	2 (2.9)	8 (13.8)
Medium	55 (46.2)	46 (39.0)	36 (51.4)	22 (37.9)
High	55 (46.2)	45 (38.1)	32 (45.7)	28 (48.3)
BMI (kg/m^2^)	25.9 ± 4.4	26.5 ± 5.0	25.4 ± 4.0	25.6 ± 4.2
Postmenopausal	48 (40.3)	36 (30.5)	27 (38.6)	17 (29.3)
Cancer-related factors				
Tumor site				
Breast cancer	102 (85.7)	102 (86.4)	59 (84.3)	51 (87.9)
Colon cancer	17 (14.3)	16 (13.6)	11 (15.7)	7 (12.1)
Radiotherapy				
Yes	74 (62.2)	71 (60.2)	46 (65.7)	34 (58.6)
No	45 (37.8)	47 (39.8)	24 (34.3)	24 (41.4)
Tumor receptor status				
Triple negative	24 (20.2)	12 (10.2)	13 (18.6)	6 (10.3)
Her2+, ER+ or PR+	11 (9.2)	18 (15.3)	6 (7.1)	10 (17.2)
Her2+, ER– and PR–	10 (8.4)	2 (1.7)	9 (12.9)	–
Her2–, ER+ or PR+	57 (47.9)	70 (59.3)	32 (45.7)	35 (60.3)
Tissue expander	9 (7.6)	10 (8.5)	5 (7.1)	5 (8.6)
	Median	Median	Median	Median
Moderate-to-high intensity total PA performed before diagnosis (min/week)	485 [240–975]	600 [300–1440]	450 [233–1005]	720 [375–1540]
Moderate -to-high intensity leisure and sport PA levels performed before diagnosis (min/week)	180 [50–375]	173 [60–330]	180 [60–315]	180 [60–345]

Continuous variables are presented as mean ± SD or as median and interquartile range, whereas dichotomous or categorical variables are presented as *n* (%)

*BMI* body mass index, *ER* estrogen receptor, *PR* progesterone receptor, *PA* physical activity, *SD* standard deviation

### Outcomes

#### Fatigue

**Fig. 2:**
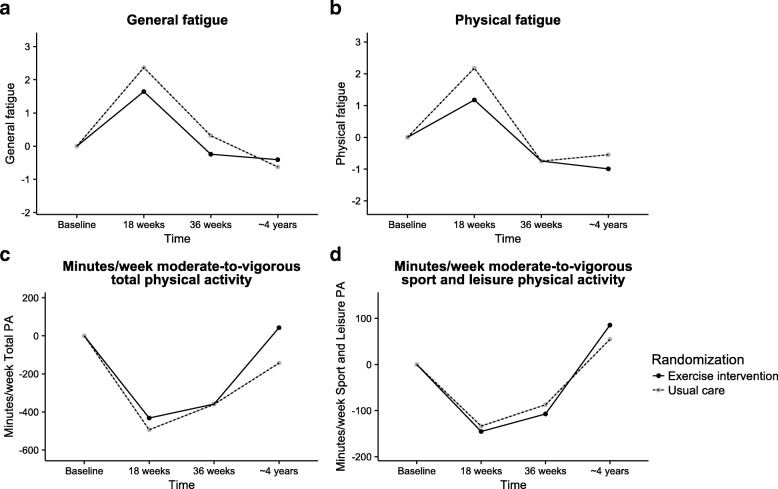
Effect of the exercise intervention on fatigue and physical activity levels over time. Intention-to-treat mixed linear regression models were used to model absolute changes in **a** general fatigue, **b** physical fatigue, **c** total physical activity, and **d** sport and leisure-time physical activity from baseline to 18 weeks, 36 weeks, and 4 years for all breast and colon cancer patients

The effects of the 18-week aerobic and resistance exercise intervention on fatigue have been published previously [6, 7]. In short, the exercise intervention showed positive effects on physical fatigue immediately after the program ending (18 weeks). At 36 weeks, this effect was no longer statistically significant (Fig. 2).

At 4 years post-baseline, only the dimension physical fatigue tended to be lower in the intervention group compared to the usual care group (− 1.13, 95% CI − 2.45, 0.20, ES = 0.22). Compared to baseline, 4-year physical fatigue did not significantly differ in the intervention group (− 1.00, 95% CI – 2.17, 0.18) and the usual care group (− 0.55, 95% CI – 1.79, 0.70). No significant differences were found for the other dimensions of fatigue (Table 2).

**Table 2:** Long-term effects of exercise on fatigue and physical activity among colon and breast cancer patients

		Baseline		Baseline to 4 years post-intervention		
				Within-group differences	Between-group differences	
		Mean	SD	Mean [95% CI]	Mean [95% CI]	Effect size
Multidimensional Fatigue Inventory (MFI)						
General fatigue	I	10.24	4.92	−0.41 [−1.52, 0.71]	−0.38 [−1.63, 0.86]	0.08
	UC	10.67	4.80	−0.63 [− 1.81, 0.56]	Reference	
Physical fatigue	I	9.92	5.12	−1.00 [−2.17, 0.18]	− 1.13 [−2.45, 0.20]	0.22
	UC	10.54	5.01	−0.55 [−1.79, 0.70]	Reference	
Mental fatigue	I	9.77	5.33	−0.23 [−1.40, 0.94]	− 0.30 [− 1.64, 1.05]	0.06
	UC	9.96	5.20	−0.24 [−1.48, 1.00]	Reference	
Reduced activity	I	8.18	4.11	−0.38 [−1.28, 0.53]	− 0.17 [− 1.23, 0.90]	0.04
	UC	8.91	4.02	−0.98 [−1.94, –0.02]*	Reference	
Reduced motivation	I	8.36	5.51	0.72 [−0.41, 1.85]	−0.18 [− 1.15, 0.79]	0.03
	UC	9.17	5.83	0.40 [−0.81, 1.61]	Reference	
Short Questionnaire of Assess Health-enhancing physical activity (SQUASH)						
Total physical activity	I UC	583.91 627.31	658.73 637.11	43.22 [− 102.13, 188.57] − 143.77 [− 298.43, 10.89]	141.46 [1.31, 281.61]* Reference	0.22
Sport and leisure physical activity	I UC	220.08 227.34	269.27 259.91	85.18 [30.65, 139.72]* 54.67 [−3.75, 113.09]	20.41 [−45.54, 86.36] Reference	0.08

*I* exercise intervention group, *UC* usual care group, *SD* standard deviation, *CI* confidence interval

*Statistically significant (*p* < 0.05)

Between-group effects were assessed using mixed models including the measurements obtained at 18 and 36 weeks and 4 years, adjusted for the value of the outcome variable at baseline as well as for stratification factors: age, radiotherapy, hospital, and tumor site. Within-group effects were assessed using mixed models including the measurements obtained at baseline, 18 and 36 weeks, and 4 years, adjusted for stratification factors: age, radiotherapy, hospital, and tumor site

Baseline results and within-group differences were based on participants having baseline measurements. Between-group differences were based on participants having measurements at 18 weeks, 36 weeks, or 4 years

#### Physical activity

The effects of the 18-week exercise intervention on physical activity levels have not been published previously. In short, moderate-to-vigorous total physical activity levels as well as leisure-time and sport physical activity levels significantly decreased from pre-diagnostic to 18 weeks (post-intervention) and 36 weeks post-baseline in both study arms (i.e., during adjuvant treatment) (Fig. 2). Four years post-baseline, participants resumed their pre-diagnostic physical activity levels. Compared to baseline, combined moderate-to-vigorous leisure-time and sport physical activity levels in the intervention group were significantly higher 4 years post-baseline (85.18 min/week, 95% CI 30.65, 139.72) (Table 2). Moreover, compared to the usual care group, participants in the intervention group reported significantly higher moderate-to-vigorous total physical activity levels (141.46 min/week, 95% CI 1.31, 281.61, ES = 0.22) 4 years post-baseline. Moderate-to-vigorous leisure-time and sport physical activity did not differ significantly (20.41 min/week, 95% CI – 45.54, 86.36, ES = 0.08).

Results for fatigue and physical activity in breast cancer participants only were attenuated, but in general comparable to results for the total group (Table 3).

**Table 3:** Long-term effect of exercise on fatigue and physical activity among breast cancer patients only

		Baseline		Baseline to 4 years post-intervention		
				Within-group differences	Between-group differences	
		Mean	SD	Mean [95% CI]	Mean [95% CI]	Effect size
Multidimensional Fatigue Inventory (MFI)						
General fatigue	I	9.28	6.08	0.13 [−1.11, 1.38]	−0.26 [− 1.65, 1.12]	0.04
	UC	10.01	6.11	−0.48 [−1.78, 0.82]	Reference	
Physical fatigue	I	9.05	6.32	−0.53 [−1.83, 0.76]	−1.05 [−2.53, 0.43]	0.17
	UC	10.01	6.35	−0.33 [−1.68, 1.02]	Reference	
Mental fatigue	I	9.32	6.64	0.05 [−1.24, 1.33]	−0.09 [− 1.59, 1.41]	0.01
	UC	9.77	6.68	−0.28 [−1.62, 1.06]	Reference	
Reduced activity	I	7.04	5.07	−0.21 [−1.21, 0.79]	0.17 [−1.02, 1.36]	0.03
	UC	7.62	5.09	−0.95 [−1.98, 0.09]	Reference	
Reduced motivation	I	7.92	6.42	0.51 [−0.73, 1.74]	−0.20 [−1.27, 0.87]	0.03
	UC	8.42	6.71	0.39 [−0.91, 1.70]	Reference	
Short Questionnaire to Assess Health-enhancing physical activity (SQUASH)						
Total physical activity	I UC	640.55 750.38	794.85 792.05	36.76 [− 125.46, 198.98] − 167.21 [− 336.75, 2.32]	117.70 [−40.61, 276.02] Reference	0.15
Sport and leisure physical activity	I UC	211.59 217.57	332.60 332.04	93.91 [32.33, 155.49]* 56.71 [−8.03, 121.45]	33.66 [−40.26, 107.57] Reference	0.10

*I* exercise intervention group, *UC* usual care group, *SD* standard deviation, *CI* confidence interval

*Statistically significant (*p* < 0.05)

Between-group effects were assessed using mixed models including the measurements obtained at 18 and 36 weeks and 4 years, adjusted for tumor receptor status and the value of the outcome variable at baseline as well as for stratification factors: age, radiotherapy, hospital, and tissue expander. Within-group effects were assessed using mixed models including the measurements obtained at baseline, 18 and 36 weeks, and 4 years, adjusted for tumor receptor status as well as for stratification factors: age, radiotherapy, hospital, and tissue expander

Baseline results and within-group differences were based on participants having baseline measurements. Between-group differences were based on participants having measurements at 18 weeks, 36 weeks, or 4 years

## Discussion

Cancer-related fatigue is a common side effect of chemotherapy. Exercise during chemotherapy might be a promising strategy for minimizing treatment-related side effects, both short term and long term [4, 9, 11, 22]. The present study assessed long-term effects of the PACT exercise intervention on fatigue and physical activity levels in patients with breast or colon cancer approximately 4 years after enrollment in the original PACT study. Breast and colon cancer patients who participated in the 18-week exercise intervention showed non-significant lower levels of physical fatigue and significant higher levels of physical activity approximately 4 years post-baseline.

In the original PACT study, physical fatigue increased significantly less in the intervention group compared to the control group during the 18-week exercise intervention [6]. Slightly lower levels of physical fatigue in participants in the intervention group were still observed after 4 years, although this was not statistically significant. This is probably due to the slightly reduced power, since only a sample of all eligible PACT participants participated in the measurements 4 years post-baseline. Nonetheless, the ES of the original randomized controlled PACT study was of comparable magnitude (ES = 0.27 18 weeks (post-intervention) vs. ES = 0.22 4 years post-baseline). So far, no other studies have investigated the long-term effects, i.e., several years post-intervention, of exercise during cancer treatment on fatigue. Since fatigue is known to be a long-lasting side effect of cancer treatment, it is important to develop interventions that reduce fatigue both in the short and long terms. More research is needed to confirm our indicative finding that exercising during chemotherapy might be a promising strategy for minimizing fatigue in the long term.

Although physical activity levels during the PACT intervention period did not differ between groups, at 4-year follow-up, participants who were randomized to the intervention group reported higher moderate-to-vigorous physical activity levels than participants randomized to the usual care group. This is a favorable result, since the PACT study was designed to promote maintenance of a physically active lifestyle by incorporating cognitive behavioral principles of Bandura’s social cognitive theory [8]. Also, in addition to the PACT exercise intervention, participants were encouraged to be physically active for at least 30 min on at least 3 other days. This recommendation might have made them more aware of the importance of integrating physical activity in daily life. According to Cohen, the ES of 0.22 can be indicated as a small difference; however, given the beneficial effects of physical activity on treatment-related side effects and prognosis, every gain in moderate-to-high intensity physical activity might be clinically relevant.

The observed effects of an exercise intervention on subsequent higher physical activity levels are comparable to results of Mutrie et al. [14], who also performed a long-term follow-up. The study included 203 patients with breast cancer in a 12-week supervised group exercise program starting during treatment for early stage breast cancer [14]. Higher leisure-time physical activity levels were observed in the intervention group compared to the control group 5 years after the intervention. The present study found higher total physical activity levels in the intervention group compared to the usual care group. In addition, they found that those who maintained a physically active lifestyle 5 years after cancer treatment still benefit in terms of increased quality of life and lower levels of depression [14]. The PACThe trial, which offered a 2-week physical and educational intervention to patients with breast cancer post-chemotherapy, found a significant improvement in breast cancer survivors’ quality of life at 5-year follow-up [23]. Results of these studies suggest that, for both breast and colon cancer survivors, engaging in exercise during chemotherapy and maintaining a physically active lifestyle into survivorship might be important for enhanced well-being in the long term. In order to optimize the long-term benefits of physical activity, more research is needed to unravel the best ways to support cancer survivors to maintain a regular exercise routine.

Our results should be viewed within the context of several strengths and limitations. Strong features of this study are the randomized design of the original PACT study, the long-term follow-up measurements 4 years post-baseline, and the intention-to-treat analyses. The present study also suffered some limitations. Only 65% of PACT participants responded, which was higher compared to the response rate in the study of Mutrie et al. [14] (42%). Nonetheless, baseline characteristics of participating patients did not significantly differ from characteristics of non-participating eligible patients. Participants in this study reported, on average, high pre-diagnostic physical activity levels and, therefore, might not be the ones who benefited from this exercise program the most. In the current study, due to the small number of patients with colon cancer, we analyzed our data for breast and colon cancer patients together, although benefits resulting from exercise may vary depending on treatment modality and tumor type. In previous analyses, a significant effect of the intervention on physical fatigue was observed at 18 weeks for both sites, but the effect was larger for colon cancer patients [6, 7]. Another limitation of our study includes the reliance on self-reported measures of physical activity, since these measures are prone to over-reporting. Objective measurement of physical activity would overcome this limitation and could provide a more valid estimate of physical activity in future studies [24, 25]. Nonetheless, subjectively measured physical activity using the SQUASH has been shown to be reliable [19], and we do not expect differential over-reporting in this 4-year follow-up. Finally, the lack of detailed information on patients who refused participation might have hampered the generalizability of the results.

## Conclusion

This study shows that an 18-week exercise intervention offered in daily clinical practice and started during early adjuvant treatment including chemotherapy has beneficial long-term effects. These beneficial effects include significantly higher levels of moderate-to-vigorous total physical activity levels and non-significant lower physical fatigue levels 4 years post-baseline. These results emphasize that facilitation of physical activity during cancer treatment may enhance health outcomes in both the short and long terms.

## Abbreviations

CI: Confidence interval
ES: Effect size
MET: Metabolic equivalent
MFI: Multifactorial Fatigue Inventory
PACT: Physical Activity during Cancer Treatment
RCT: Randomized controlled trial
SQUASH: Short Questionnaire to Assess Health-enhancing physical activity
SD: Standard deviation
